# Incorporation of *Limosilactobacillus fermentum* UCO-979C with Anti-*Helicobacter pylori* and Immunomodulatory Activities in Various Ice Cream Bases

**DOI:** 10.3390/foods11030333

**Published:** 2022-01-25

**Authors:** Cristina Paucar-Carrión, Marcela Espinoza-Monje, Cristian Gutiérrez-Zamorano, Kimberly Sánchez-Alonzo, Romina I. Carvajal, Cristian Rogel-Castillo, Katia Sáez-Carrillo, Apolinaria García-Cancino

**Affiliations:** 1 Laboratory of Bacterial Pathogenicity, Department of Microbiology, Faculty of Biological Sciences, Universidad de Concepción, Concepción 4070386, Chile; c.paucar.3@gmail.com (C.P.-C.); marespinozamonje@gmail.com (M.E.-M.); cristian0903gutierrez@gmail.com (C.G.-Z.); kimsanchez@udec.cl (K.S.-A.); rominacarvajal@udec.cl (R.I.C.); 2 Department of Food Science and Technology, Faculty of Pharmacy, Universidad de Concepción, Concepción 4070386, Chile; crogel@udec.cl; 3 Department of Statistics, Faculty of Physical and Mathematical Sciences, Universidad de Concepción, Concepción 4070386, Chile; ksaez@udec.cl

**Keywords:** probiotics, *Limosilactobacillus fermentum* UCO-979C, *Helicobacter pylori*, anti-*H. pylori* activity, ice cream, dairy food, functional food

## Abstract

*Limosilactobacillus fermentum* UCO-979C is a probiotic strain possessing anti-*Helicobacter pylori* and immunomodulatory activity. The aim of this work was to examine if this strain maintains its probiotic properties and its viability when added to dairy-based ice creams (cookies and cream, Greek yogurt, and chocolate with brownie) or to fruit-based ice creams (pineapple and raspberry) stored at −18 °C for 90 days. The probiotic anti-*H. pylori* activity using the well diffusion test, its immunomodulatory activity was measured using transforming growth factor beta 1 (TGF-β_1_) cytokine production by human gastric adenocarcinoma (AGS) cells, and its viability was measured using the microdrop technique. Assays were performed in triplicate. The *L. fermentum* UCO-979C strain maintained strong anti-*H. pylori* activity in dairy-based ice creams and mild activity in fruit-based ice cream. The production of pro-inflammatory cytokine TGF-β_1_ on AGS cells was higher in the probiotic recovered from Greek yogurt ice cream, maintaining a viability exceeding 10^7^ colony-forming units/mL. The addition of the probiotic to ice creams did not significantly influence the physicochemical properties of the product. These data show the great potential of the *L. fermentum* UCO-979C strain in producing probiotic dairy-based and fruit-based ice creams.

## 1. Introduction

*Helicobacter pylori* infection is considered a major factor in the development of 44.4% of gastric cancer, killing nearly 800,000 people worldwide in 2018 [1]. This bacterium is considered a type I carcinogen by the World Health Organization (WHO), and it is closely related to the development of gastritis, duodenal ulcers, and MALT lymphoma [2]. Due to its association with these pathologies, it is necessary to eliminate this bacterium from the human body by means of pharmacological treatments consisting of antibiotics, such as the administration of clarithromycin, metronidazole, or levofloxacin combined with a proton pump inhibitor for 7 to 14 days [3]. A study reported that between 15 and 31% of patients were resistant to drug treatments due to the lack of patient observance to treatments because they produce adverse effects and their cost is high [4]. It has been reported that the combination of established pharmacological treatments together with probiotics would modulate certain discomforts, such as a reduction in adverse effects [5,6,7,8], achieving compliance of the patient to the pharmacological therapy. Most probiotics are members of the lactic acid bacteria group, and according to the Food and Agriculture Organization (FAO), they are defined as living microorganisms that, when administered in sufficient quantities, confer health benefits [9]. Probiotic strains to be administered must maintain their viability, resist adverse environments during food processing and storage prior to consumption, and retain their properties unaltered [10]. That is why ice cream has been considered an ideal vehicle to deliver probiotics because its neutral pH maintains the metabolic activities of lactic bacteria, it is highly accepted by different populations [11], and it could provide extra nutritional value by having probiotics in its formulation [12]. However, freezing and thawing processes could damage the metabolic processes and cause the death of lactic bacteria [13]. Several studies have shown that the probiotic strain originally named *Lactobacillus fermentum* UCO-979C, now classified as *Limosilactobacillus fermentum* UCO-979C, isolated in 2007 from a gastric biopsy, has anti-*H. pylori* activity [14]. This strain also is a producer of lactic acid and affects the urease enzymes produced by *H. pylori* [15]. Furthermore, an in vitro study demonstrated that the *L. fermentum* UCO-979C strain decreased the production of cytokines and inflammatory chemokines in a human gastric adenocarcinoma (AGS) and in the THP-1 cell lines and that it increased the production of immunoregulatory cytokines, such as TGF-α, in those same cell lines [16]. An in vivo study using Mongolian gerbils showed that the administration of the *L. fermentum* UCO-979C strain was able to colonize the stomach of this animal model and to decrease the count of yjr *H. pylori* SS1 pathogenic strain [17]. Additionally, in an in vivo trial using Swiss mice, it was found that the exopolysaccharides produced by *L. fermentum* UCO-979C are involved in the immunomodulatory effect, interacting with certain receptors capable of modulating the production of inflammatory mediators [18].

Currently, to our knowledge, there are no commercial ice creams with anti-*H. pylori* or immunomodulatory characteristics. Hence, we are interested in investigating the behavior of the probiotic *L. fermentum* UCO-979C strain when added to various ice cream bases. In particular, the aim of this study was to determine whether the viability, anti-*H. pylori* activity, and immunomodulatory activity of the *L. fermentum* UCO-979C strain would be maintained after it was added to ice cream and whether it would influence the physicochemical properties of ice cream stored at −18 °C for 90 days.

## 2. Materials and Methods

### 2.1. Bacterial Strain and Growth Conditions

All bacterial strains used in this study were available at the Laboratory of Bacterial Pathogenicity, Department of Microbiology, University of Concepcion, Chile.

The probiotic strain *L. fermentum* UCO-979C of human gastric origin was used. It was cultured using Mann–Rogosa Sharpe (MRS) broth (Difco, Wokingham, UK) under microaerobiosis at 37 °C for 24 h followed by culturing in MRS agar (BD Difco, Sparks, MD, USA) under similar conditions for another 24–48 h. The *H. pylori* J99 strain (also known as ATCC 700824), originally obtained from American Type Culture Collection (ATCC) (Manassas, VA, USA), was cultured in Columbia blood agar base (CA) (Oxoid, Basingstoke, UK) supplemented with 5% horse blood and selective supplement DENT (Oxoid, Basingstoke, UK) in a microaerobic atmosphere (10% CO_2_, 5% O_2_, 85% N_2_) at 37 °C for 72–96 h. To confirm the purity of *H. pylori* J99 culture, Gram staining and urease, catalase, and oxidase tests were performed.

The biomass of the probiotic *L. fermentum* UCO-979C strain was produced at the premises of the Processes and Foods Laboratory, Faculty of Chemical Engineering, University of Concepcion, Concepción (Chile). The culture medium contained whey cheese, lactase, casein peptone, yeast extract, potassium dihydrogen phosphate, sodium acetate, and tween 80, and its patent number is 1940–2005 (National Institute of Industrial Property—INAPI, Santiago, Chile), its pH was 6.8. The culture was maintained under aerobic conditions at 37 °C in a 15 L fermenter. The biomass generated was filtered using a sterile 0.22 µm filter and a vacuum pump model FD-10 Bench-top type (Xiangyi, Hunan, China), resuspended in 20% skim milk, frozen at −45 °C, and freeze-dried in a 50 L model BK-FD10PT freeze dryer (Biobase, Shandong, China). The biomass was stored at 4 °C in the darkness until use. The count of the strain in the milk powder was 10^10^ colony-forming units CFU/g of lyophilized powder, which was kept at 4 °C in the darkness until use.

### 2.2. Ice Cream Production

The ice creams were supplied by the company Sociedad Comercial Acuña Aguayo Limitada (San Pedro, Biobío Region, Chile), using five varieties of ice cream: two natural fruit varieties (raspberry and pineapple) and three dairy varieties (chocolate with brownie, cookies and cream, and Greek yogurt). The manufacturer kept the preparation of the different varieties of the ice creams confidential. Each ice cream variety was divided into two portions: one of them was supplemented with the freeze-dried probiotic *L. fermentum* UCO-979C strain at a final concentration of 10^8^ CFU/mL, and the second portion, lacking the probiotic, was used as the control. The mixtures were deposited in stainless steel molds placed in a freezing machine. The portions with or without probiotic were cooled to 4 °C for 12 h, then deposited in stainless steel molds, and frozen at −20 °C. The final product (ice cream) was finally stored at −18 °C. For each assay, three independent replicates of the portions with or without probiotic for each one of the five varieties of ice cream were analyzed and frozen at −20 °C. The final product (ice cream) was finally stored at −18 °C. For each assay, three independent true replicates of the portions with or without probiotic for each one of the five varieties of ice cream were analyzed.

### 2.3. Viability of L. fermentum UCO-979C in Ice Cream

The probiotic strain was recovered by dissolving 2 g of ice cream in 20 mL of sterile water. From this mixture, 10 µL were added to 900 µL of 0.9% saline solution; then, two serial dilutions prepared. The dilutions were used to count the CFU using the microdrop technique following the protocol of Sanhueza et al. [19], as described next. Fifteen microliters of these dilutions were deposited on an MRS agar in drop form, which was incubated at 37 °C under microaerobic conditions (10% CO_2_) for 24 h. The counts were expressed as log (CFU/mL).

The colonies were counted for each of the dilutions. The CFU count was performed at day 15 (time 0 = T0), corresponding to the day when the ice creams were transferred by the manufacturer to our laboratory. All analyses were performed in triplicate for each variety of ice cream flavors.

### 2.4. Anti-H. pylori Activity of the Probiotic Strain Recovered from Ice Cream

The anti-*H. pylori* activity of the probiotic *L. fermentum* UCO-979C strain was determined using the well diffusion test according to Vega et al. [20], with minor modifications. The *H. pylori* J99 strain was seeded at a concentration of approximately McFarland 2 scale (6 × 10^8^ CFU/mL) on CA agar supplemented with 5% horse blood. The agar was punctured with 6 mm glass Pasteur pipettes (Corning BV, Tewksbury, MA, USA). The wells were filled with 50 µL of non-citrate MRS (negative control), 50 µL of 0.9% saline (negative control), 50 µL of non-citrate MRS plus *L. fermentum* UCO-979C (0.5 McFarland (1.5 × 10^8^ CFU/mL)) or the lyophilized strain (positive control), 50 µL of non-citrate MRS plus *L. fermentum* UCO-979C (0.5 McFarland (1.5 × 10^8^ CFU/mL)) obtained from the different varieties of ice creams, 50 µL of pellets, and 50 µL of the supernatant obtained from a *L. fermentum* UCO-979C culture in MRS previously centrifuged at 3500× *g* during 1 min. Then, the dishes were incubated at 37 °C at 10% CO_2_ for 72 h. This assay was performed in triplicate, sampling each ice cream variety after 90 days of storage at −18 °C. The probiotic strain was obtained from the same dilution of ice cream in distilled water that was seeded on MRS agar and incubated at 37 °C with 10% CO_2_ in an incubator (Thermo Scientific, Waltham, MA, USA) for 24 h. Then, a colony was taken and reseeded in MRS broth and (Difco, Wokingham, UK) without citrate and incubated at 37 °C with 10% CO_2_ for 15 h in the same equipment. To determine the size of the inhibition halo, the criteria described by Gaudana et al. [21] was used, where a halo >5 mm = + + +, implies a very strong inhibition; 5 mm and >2 mm = + +, a strong inhibition; 2 mm and >1 mm = +, with a slight inhibition; and ≤1 mm = +/−,without inhibition.

### 2.5. Immunomodulatory Activity of the Probiotic Strain Recovered from Ice Cream

Additionally, the immunomodulatory activity was determined as described by García et al. [16]. The effect on the production of pro-inflammatory cytokine TGF-β1 on AGS cells by the *L. fermentum* UCO-979C strain was measured after 90 days of storage of the ice creams at −18 °C using the ELISA technique. *L. fermentum* UCO-979 was obtained from the lyophilizate (control sample) and from the ice cream sample (fruits varieties: raspberry and pineapple; dairy varieties: chocolate with brownie, cookies and cream, and Greek yogurt). The probiotic strain was seeded on MRS agar and incubated for 18 h at 37 °C in the presence 10% CO_2_. The AGS cells were seeded in 24-well plates (Thomas Scientific, Swedesboro, NJ, USA) at a concentration of 7 × 10^4^ cells/mL in (DMEM Thermo Scientific, Waltham, MA, USA) supplemented with 10% *v*/*v* of inactivated fetal bovine serum (FBS) (Biological Industries, Cromwell, CT, USA), 100 U/mL of penicillin, and 100 μg/mL of streptomycin (Corning BV, Tewksbury, MA, USA). The plates were incubated for 24 h at 37 °C and at 5% CO_2_ until reaching an 80 to 90% confluence. The probiotic obtained was then suspended in DMEM medium without antibiotics at a concentration of 10^5^ or 10^7^ CFU/mL and added to the 24 well plate containing the AGS cells (ATCC, Manassas, VA, USA) at a concentration of 7 × 10^4^ cells/mL and incubated for 24 h at 37 °C and in the presence of 5% CO_2_.

The supernatants of the AGS cells plus the probiotic *L. fermentum* were then recovered, and the cytokines were measured in duplicate using the TGF-β_1_ cytokine kit Invitrogen Human/Mouse TGF-β_1_ Uncoated (Thermo Scientific, Waltham, MA, USA) at a wavelength of 450 nm using an Infinite M200 PRO equipment (TECAN, Männedorf, Switzerland).

### 2.6. Chemical and Physical Analysis of Ice Cream

Proximal chemical analysis of the ice creams was performed using the methods described by Latimer G.W [22].

#### 2.6.1. Chemical Analysis

Moisture was determined by the method AOAC 931.04, ash was determined by the gravimetric method (AOAC 945.46–942.05), and total protein was determined by the Kjeldahl method with a conversion factor of 6.38 (AOAC 928.08–930.33) [22]. The percentage of fat was measured by the Gerber method (AOAC. 952.06–989.05); the crude fiber was measured by the method AOAC 985.29, and the total sugars were measured by the methods AOAC 31.037 and 31.040 [22]. The titratable acidity was determined by the volumetric method, adding phenolphthalein over NaOH and expressed as percentage of lactic acid (AOAC 969.21) [22]. The pH values were read with a pH meter Model 350 (Bante, Shanghai, China). All of these procedures were performed, in triplicate, for each type of ice cream.

#### 2.6.2. Physical Analysis

The melting speed and the first drop fall time was performed according to the methodology proposed by Abd El-Ramh [23], with certain modifications, at a controlled temperature of 24 ± 2 °C. Each ice cream sample was placed on a wire mesh with an opening of 2 mm, and it was allowed to melt in a beaker placed on an analytical balance (KERN, PB) for 60 min, evaluating the melted ice cream mass every 2 min, monitoring it through a chronometer, after the fall of the first ice cream drop. The ice cream melting rate was calculated from the slope, which is the ratio between the grams of ice cream melted by time [24].

The color of probiotic and control ice cream stored at −18 °C for 90 days was measured with a handheld colorimeter, model Cs-280 (CHNspec, Hangzhou, China) [25]. The result was reported by the CIE L*a*b* system, where the L* coordinate is the brightness, with L = 0 (black) and L = 100 (white); a* corresponds to positive values reflecting red and negative values reflecting green; and for b* values, the positive values correspond to yellow and negative values correspond to blue, as well as the L*C*h system, where the C* coordinate, called chroma, represents intensity or purity and h* is hue, which is the color shade angle [26]. The total color difference index (ΔE) is determined by calculating the following equation:

∆E = [(ΔL*)^2^ + (Δa*)^2^ + (Δb*)^2^]^1/2^

where ∆E corresponds to the difference between the color of the control sample (L* 1, a* 1, and b* 1) and the probiotic supplemented ice cream sample (L* 2, a* 2, and b* 2) for each variety [24,27]. A value greater than 2 indicates a change in color [27].

This procedure was performed for each variety of ice cream in triplicate.

### 2.7. Statistical Analysis

The statistical analysis was performed using the SPSS software version 24.0 (IBM Company, Armonk, NY, USA). The values of the physicochemical determinations were expressed as means and standard deviation. Bacterial counts were analyzed by the Student’s *t*-test for independent samples (two groups) or by a two-way analysis of variance (ANOVA); in the latter case, the Tukey multiple comparison test was applied. In all cases, a significance level of 0.05 was used.

## 3. Results

### 3.1. Viability of L. fermentum UCO-979C in Ice Cream

**Figure 1:**
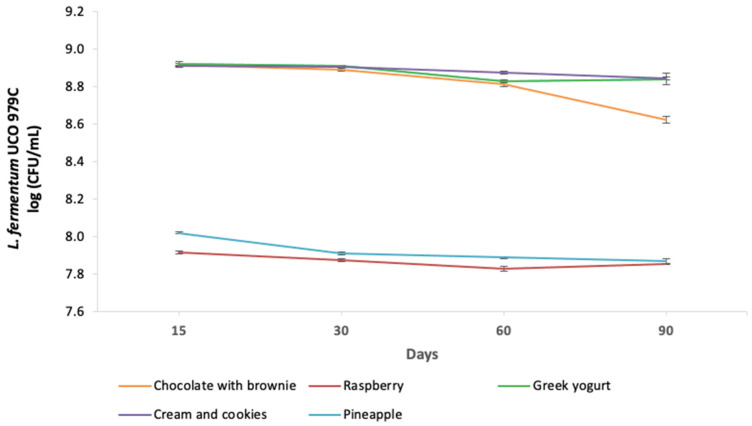
Viability of the probiotic *L. fermentum* UCO-979C strain in different ice cream bases during 90 days of storage at −18: day 15 (T0), the time of storage of the ice creams by the manufacturer and the day they were delivered to the laboratory; day 30 (T1); day 60 (T2); and day 90 (T3).

The viability of the probiotic *L. fermentum* UCO-979C strain in the different fruit-based and dairy-based ice cream bases containing the probiotic strain showed that both types of ice cream matrices allowed for the probiotic to retain its viability, with counts above 10^7^ CFU/mL during the three months of its storage at −18 °C (Figure 1). When comparing the behavior of *L. fermentum* UCO-979C strain in ice creams of the same basis, among the dairy-based ice creams, it was possible to observe a significant viability reduction in the probiotic in chocolate with brownie ice cream after 60 days and 90 days of storage at −18 °C when compared with the cookies and cream and the Greek yogurt ice creams (Table 1). With respect to fruit-based ice creams, a significant reduction in the viability of the probiotic was observed in the case of pineapple ice cream on day 30 of storage, which remained without significant changes until day 90 of storage at −18 °C (Table 1).

### 3.2. Anti-H. pylori Activity of L. fermentum UCO-979C

When the anti-*H*. *pylori* activity of the probiotic *L. fermentum* UCO-979C strain was assayed after 90 days of storage of the ice creams at a temperature of −18 °C, the supernatant of the probiotic strain isolated from the dairy-based probiotic ice creams showed strong inhibition (5 mm and >2 mm = ++ halo) in the growth of *H. pylori* J99, similar to that of the total culture of the probiotic strain, while the inhibition caused by the pellet was not strong for *H. pylori* J99. It should be noted that the greatest inhibition of *H. pylori* growth was observed in the Greek yogurt-based ice cream. In the fruit-based ice creams supplemented with probiotics, the probiotic inhibition activity was mild in the supernatant and the culture medium, while for the pellet, the inhibition was considered null. When analyzing the lyophilized probiotic strain that was not added to any ice cream base or subjected to low temperatures, its anti-*H. pylori* activity was mild (2 mm and >1 mm = + halo) in the supernatant as in the culture medium and null in the pellet (Table 2).

### 3.3. Immunomodulatory Activity of L. fermentum UCO-979C

**Figure 2:**
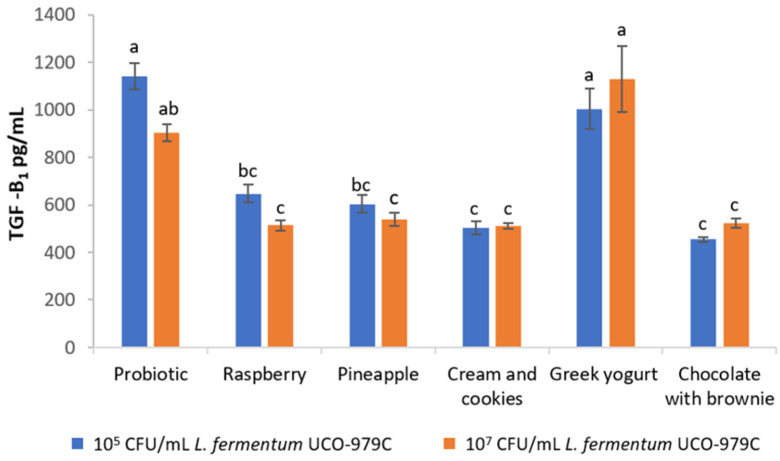
Production of TGF-β_1_ cytokines by gastric epithelial cells (AGS) induced by the *L. fermentum* UCO-979C strain obtained from the different ice cream bases at 10^5^ and 10^7^ CFU/mL after 90 days of storage at −18 °C. The name of each ice cream corresponds to the *L. fermentum* UCO-979C strain obtained from each one of the flavors, and probiotic (control) corresponds to *L. fermentum* UCO-979C obtained directly from the lyophilizate. Means with different superscript letters are significantly different (*p* < 0.05).

The effect on the production of pro-inflammatory cytokines TGF-β1 in AGS cells by *L. fermentum* UCO-979C strain was measured after 90 days of storage of the ice cream at −18 °C using the ELISA technique. When comparing the results obtained with both concentrations of *L. fermentum* UCO-979C, 10^5^ and 10^7^ CFU/mL, obtained from the different varieties of ice creams (fruit-based and dairy-based), it was possible to observe that both treatments were able to retain the immunomodulatory activity (Figure 2) with no significant differences between them. Moreover, Figure 2 shows that a concentration of 10^5^ CFU/mL of the probiotic strain obtained directly from the lyophilizate showed cytokine production without significant difference when compared with the Greek yogurt ice cream (*p* > 0.05). Therefore, this ice cream flavor caused no alterations on the immunomodulating activity of *L. fermentum* UCO-979C when compared with the strains obtained from the other ice cream flavors.

Regarding the fruit-based ice creams, it was observed that the cytokine production was less than that of the strain obtained directly from the lyophilizate or from the Greek yogurt ice cream; nevertheless, it still remained high compared with the other dairy-based ice creams (*p* < 0.05) (Figure 2).

### 3.4. Physicochemical Analysis

#### 3.4.1. Proximal

Proximal analysis was performed on the control and probiotic ice cream after 30 days of storage (Table 3 and Table 4). It was possible to observe that, in all ice creams, both fruit-based and dairy-based ice creams, there was a decrease in the pH when compared with the control. Additionally, the probiotic dairy-based ice cream, such as chocolate with brownie, had a higher percentage of fat and pH when compared with the same ice cream without the probiotic. On the other hand, there was a lower percentage of moisture in Greek yogurt ice cream and a lower pH in probiotic cookies and cream. In the fruit ice creams, the raspberry with probiotic showed a variation in the percentage of protein, pH, and lactic acid, while the pineapple probiotic ice cream had a higher percentage of lactic acid and pH.

The probiotic ice creams had a lower or faster first-drop compartment, with a longer melting time compared with ice creams without the probiotic; this with statistically different (*p* < 0.05). The pineapple probiotic ice creams showed lower resistance to the first-drop process, with a lower melting speed (*p* < 0.05). Probiotic dairy-based ice creams, such as chocolate with brownie, and cookies and cream had higher or faster melting speeds (*p* < 0.05) (Figures S1 and S2).

#### 3.4.2. Color

The analysis of color is a control measure in food and provides organoleptic information about the product, allowing us to appreciate and give a criterion upon which to choose a food product. The probiotic fruit ice cream had a color change with significant differences (*p* < 0.05), with a color variation greater than that of the control ice cream. The raspberry ice cream luminosity (L*) increased, with less intensity and a red with yellow tendency. In the case of pineapple ice cream, the luminosity values decreased, tending to become more saturated than the control ice cream; however, its color remained yellow (Table 5).

In dairy-based ice creams, such as cookies and cream, and chocolate with brownie, the brightness remained unchanged during the 90 days of storage, but in the Greek yogurt, the brightness increased significantly (*p* < 0.05). As for the saturation of the cookies and cream and the Greek yogurt ice creams, it decreased significantly (*p* < 0.05) every month (Table 5).

## 4. Discussion

When comparing the viability of the probiotic strain in dairy-based ice creams with those in fruit-based ice creams, a significant difference was observed; both matrices supported the viability of *L. fermentum* UCO-979C, in the rage of 10^7^ CFU/mL, complying with what is imposed by Technical Standard No. 191 of the Chilean Ministry of Health. This regulation requires that foods containing strains of live bacilli must contain at least 10^7^ CFU/mL of probiotic per gram of product [28]. The results obtained in this study are similar to those reported by Chiquetti et al. [29], who made probiotic ice cream with the *Lactobacillus acidophilus* La-5 strain together with β-galactosidase, which hydrolyses lactose, and they obtained probiotic counts above 10^7^ CFU/g during 120 days of storage of the ice cream. In this same sense, Dos Santos et al. [13] also reported that the survival of *Lactobacillus delbrueckii* UFV H2b20 was not significantly affected (*p* > 0.05) in three dairy-based ice cream formulations after processing or during storage for 40 days at −16 °C, maintaining a concentration of more than 10^8^ CFU/g.

Therefore, the results reported by the present work, supported by those of the abovementioned authors, support that dairy-based ice creams constitute a favorable environment that allows probiotic bacteria, such as *L. fermentum* UCO-979C, to retain their viability. Hence, ice cream is an ideal vehicle to deliver these microorganisms into the human diet [30,31].

The high survival of probiotic bacteria in dairy-based ice cream may be due to the high total solids level in ice cream, including fat and milk solids, which provide protection to probiotic bacteria [30]. Probiotic ice creams with a fat content ranging from 8% to 12% have been shown to provide a protective capacity to probiotic microorganisms exposed to low freezing temperatures (−23 °C to −30 °C) [32]. In the present study, the fat content of the ice creams in dairy-based ice cream ranged from 1% to 6%; therefore, the viability of the probiotic strain cannot be attributed to the presence of this fat content. However, it needs to be further studied.

Additionally, sugars (i.e., lactose and in particular sucrose), which are used as key ingredients in dairy and ice cream desserts, have also been reported to increase the viability of probiotics because they act as cryoprotectants [33]. Short-chain polysaccharides have been found to be more cryoprotective than long-chain polysaccharides. Short-chain polysaccharides considered “prebiotic compounds” (i.e., fructo-oligosaccharides) have the most beneficial effects on probiotic stability [33].

On the other hand, since fibers acts as prebiotics, the viability of *L. fermentum* UCO-979C during the 90 days of storage at −18 °C in fruit-based ice cream can be attributed to the percentage of dietary fiber in raspberry and pineapple ice creams, benefiting the growth and viability of the probiotics strains. Ayar et al. [34] determined that the survival of probiotic strains of *L. acidophilus* and *Bifidobacterium animalis* subsp. *lactis* in ice cream samples supplemented with sources of dietary fiber from different fruits act by protecting the probiotics during the storage period and by maintaining the viability of the probiotic strains in the range of 7.0 log CFU/g. Therefore, the presence of fiber may also be a factor benefiting the viability of *L. fermentum* UCO-979C in the fruit-based ice creams analyzed in our study. Other investigations, such as the one by Di Criscio et al. [35], also supported the protecting property of fibers present in fruit for probiotics demonstrating the survival of probiotic *Lactobacillus casei* and *Lactobacillus rhamnosus* strains contained in fruit-based ice creams. The results reported by these authors showed that both probiotic strains analyzed maintained viabilities between 6.5 and 6.9 log CFU/g after freezing and during the storage period at −20 °C, even if only small doses of additional fiber, such as inulin (3%), were added to the formulation [35].

The viability of *L. fermentum* UCO-979C in pineapple ice cream is consistent with that observed by Nguyen et al. [36] because the viability of probiotic strains of *Lactobacillus* incorporated in pineapple juice remained constant during the first month of storage and subsequently decreased. On the other hand, the loss of viability of bacteria in fruit-based ice cream may be the result of the freezing process, which would affect the cell wall of the probiotic due to the formation of ice crystals, temperature stress, and dehydration of the cells. Thus, the probiotic could be affected by freezing because, when a juice that will not freeze is added to the ice cream, the viability of the probiotic strain will be maintained, but when the probiotic and fruit extract mixture freezes, there will be a decrease in CFU counts [37]. This was indicated by the survival of two probiotic microorganisms at −10 °C, where the probiotic *L. acidophilus* La-5 had a survival rate of 87%, with a count of 2.1 × 10^6^ viable CFU/g and *Bifidobacterium lactis* Bb-12 at a concentration of 9.0 × 10^6^ CFU/g with a survival rate of 90% for 2 months [38]. Thus, it can be suggested that the freezing process should be slow because the cells suffer osmotic damage and longer dehydration time and because ice crystals would form, causing damage to the membrane structures [39].

With respect to the results reported here on the anti-*H. pylori* activity of *L. fermentum* UCO-979C, it was possible to observe that the matrix containing the probiotic strain had an effect on its inhibitory capacity, having a better anti-*H. pylori* effect in dairy-based ice creams. This may be due to the individual properties of each ice cream, which might increase the anti-*H. pylori* activity of the *L.* *fermentum* UCO-979C strain. Regarding anti-*H. pylori* activity in dairy-based ice cream, the greatest inhibition of the pathogenic bacterium was observed in Greek yogurt ice cream, probably because yogurt is already considered an ideal vehicle for the viability of probiotics [40]. The results of this work, using *L.* *fermentum* UCO-979C, support that asseveration (Figure 1). Moreover, there is a difference in the anti-*H. pylori* activity of *L. fermentum* UCO-979C when the inhibition assay is performed using the supernatant of the bacterial culture, showing a better anti-*H. pylori* activity in dairy-based ice creams, or the total bacterial culture. Thus far, we have been unable to find literature supporting the increased anti-*H. pylori* activity observed in the supernatant of the probiotic dairy-based ice creams. Nevertheless, since *L. fermentum* UCO-979C obtained from dairy-based ice creams was not subjected to any washing before being cultured in MRS, it is possible that small remnants of milk might have increased the *H. pylori* inhibitory activity, perhaps suggesting that the presence of bovine lactoferrin might improve the inhibition of *H. pylori* [41,42]. Regarding the *H. pylori* inhibitory capacity observed in the supernatant of the *L. fermentum* UCO-979C culture obtained from the chocolate with brownie ice cream, it is also possible that lactoferrin might also be involved in the antimicrobial activity shown by the cocoa. It has already been shown that cocoa has tannins and alkaloids, capable of penetrating the cell wall of the bacteria and inhibiting its growth [43]. Nevertheless, all of these suggestions require confirmation by future studies.

In the case of the anti-*H.* *pylori* activity of the *L. fermentum* UCO-979C culture supernatant obtained from fruit-based ice creams, it was mild when compared with the control and the supernatants obtained from dairy-based ice creams. It has been reported that antioxidants present in fruits, such as raspberry and pineapple, possess antibacterial activity and that berries are fruits rich in antioxidant and phenolic compounds capable of neutralizing the growth of *H.* *pylori* [44], thus favoring the anti-*H.* *pylori* activity of the *L. fermentum* UCO-979C strain. Chatterjee et al. [45] reported that extracts obtained from raspberry berries inhibit *H. pylori* and increase the sensitivity of this bacterium to clarithromycin. It was also reported that phenolic compounds in pineapple inhibit the growth of *H. pylori* [46]. Thus, we can suggest that it is possible that a similar effect may exist when raspberry or pineapple are combined with the probiotic strain used in the present work, an interesting issue to be addressed in future studies on this subject.

Regarding the results observed with the supernatant of the *L. fermentum* UCO-979C strain obtained directly from the lyophilizate, it showed a strong anti-*H. pylori* activity. This differs from that reported by Garcia et al. [15], where the inhibition of the probiotic strain was very strong, with a halo of inhibition >5 mm. This difference could probably occur during the freezing–drying process used to obtain the lyophilizate, where the strain could suffer a loss of activity caused by freezing and drying stress or by damage to the bacterial membrane structure [47].

The immunomodulating activity of *L. fermentum* UCO-979C has already been demonstrated by García-Castillo et al. [16], and the results obtained in the present study demonstrated that the probiotic *L.* *fermentum* UCO-979C strain retained its immunomodulatory activity, promoting the production of TGF-β_1_ in AGS cells at both concentrations assayed (10^5^ or 10^7^ CFU/mL), even after being frozen for 90 days at −18 °C. Although the probiotic retained its immunomodulating activity in dairy-based ice creams, it was possible to observe that *L. fermentum* UCO-979C strains isolated from chocolate with brownie, or cookies and cream ice creams supplemented with 10^5^ or 10^7^ CFU/mL of the probiotic caused a significant reduction in TGF-β_1_ when *L. fermentum* 979C was compared with the lyophilized strain (*p* < 0.05). In the case of Greek yogurt ice cream supplemented with 10^5^ or 10^7^ CFU/mL of the probiotic strain, there was no significant changes in the production of TGF-β_1_ when compared with the control. Therefore, since it allows us to retain both the immunomodulatory activity and the viability of this probiotic strain, Greek yogurt ice cream can be considered an ideal matrix for the administration of *L. fermentum* UCO-979C. These results coincide with those reported by Garcia et al. [16], providing evidence that the increased concentration of *L. fermentum* UCO-979C caused a higher production in TGF-β_1_ than in the other cytokines on AGS cells. In addition, studies by Yang et al. [48] also reported that the administration of yogurt with probiotics in healthy children increased the production of TGF-β_1_. As for probiotic fruit ice creams, there was a significant decrease (*p* < 0.05) in the immunomodulatory activity detected by a reduction of cytokine production.

Regarding the proximal analysis performed for the different flavors of ice creams with or without probiotic supplement in both dairy-based and fruit-based ice creams, there was a significant pH reduction (*p* < 0.05) when compared with the control ice creams (ice creams without probiotic). This may be the direct consequence of a high lactic acid production by *L. fermentum* UCO-979C [15], reducing the pH of their environment. This is consistent with the results obtained when analyzing the fruit-based ice creams, in which a significantly increase of lactic acid was also observed in the raspberry and pineapple (*p* < 0.05) ice creams. The presence of lactic acid may explain the decrease in the pH values of the ice cream matrices [15]. Despite the decrease in the pH and the increase in lactic acid in some ice creams, no important changes were observed in the nutritional components of the probiotic ice creams when compared with the control ice creams.

On the other hand, with respect to the first-drop compartment tests in probiotic ice creams, it was observed that they had a significantly lower or faster (*p* < 0.05) first-drop compartment and a longer melting time when compared with ice cream without probiotics. This is possibly due to the influence of pH, since the decrease in pH occurs with increased production of lactic acid due to the fermentation in dairy-based ice creams [49]. Salas et al. [50] determined that the *L. fermentum* UCO-979C strain synthesizes exopolysaccharides capable of forming a biofilm. Exopolysaccharides are capable of acting as stabilizers, emulsifiers, gelling agents, and cell protectors in dehydration processes. Emulsifiers have the function of increasing the resistance to melting by destabilizing the fat [51], which occurs when they bind to milk proteins and displace them. Thus, fat can have a balanced destabilization because a minor destabilization causes rapid melting and an excessive destabilization causes the structure of the ice cream to not collapse at room temperature even if the ice melts [52]. For fruit ice cream, the system of first drop and melting time decreased significantly, possibly due to the water content. When the temperature increases, the heat present in the system is eliminated; ice crystals begin to form; and an increase in temperature produces internal vibrations in the molecules, causing the ice to melt [53]. The fruit content could also influence the melting of ice cream because the fruit can limit the free movement of water molecules, as reported in Erkaya et al. [54], who showed that the addition of fruit in different concentrations increased the time of first drop and complete melting of the ice cream.

Finally, in the changes in color parameters in control ice cream (ice creams not supplemented with the probiotic strain) and ice cream supplemented with *L. fermentum* UCO-979C strain, it was possible to observe that the color changed in fruit-based ice creams. This could be the result of changes in compounds responsible for the color of fruits such as anthocyanins present in raspberries, which may suffer instability in the shade of color by increasing the pH of the storage medium. This reaction is called batochrome effect, and it is characterized by the displacement of longer wavelengths in relation to its original molecule and has a red color [55]. This could be related to the results of h* and pH in raspberry ice cream, both significantly reduced (*p* < 0.05) with respect to the control. The L*C*h* values in pineapple probiotic ice cream decreased; this is related to what Ferreira et al. [56] reported on bioactive compounds, such as carotenoids responsible for the color of these fruits, which undergo changes depending on the pH of the environment. The study by Bartolome et al. [57] showed that pineapple slices at low temperatures tend to lose their chromaticity. In the case of dairy-based ice cream, this change was probably due to the lack of homogeneity in the samples since they contained pieces of food such as cookies distributed in the ice cream.

As for the saturation of the cookies and cream and the Greek yogurt ice creams, color decreased significantly (*p* < 0.05) every month. This probably occurred due to the thin ice layer formed in the surface of the ice cream when transferred from −18 °C to room temperature, changing the appearance of the color. Since the high temperatures lead to a surface melting that causes a separation of the components of the ice cream matrix, the water crystals melt and flow. As a result, there will be more or less color concentrated on certain surfaces of the ice cream depending on the type of pigment used [58].

The color difference (ΔE) in the fruit ice cream was ΔE = 3.920 for raspberry and ΔE = 2171 for pineapple. Considering that the values for a notable difference of color magnitude should be ΔE ≥ 2, in the case of the dairy-based ice creams (cookies and cream, chocolate with brownie, and Greek yogurt), the variations had no major significant changes [24]. This suggests that the dairy-based ice creams could be considered as more appropriate ice cream bases to incorporate the probiotic strain than fruit-based ice creams.

## 5. Conclusions

The results obtained in the present work demonstrate that *L. fermentum* UCO-979C strain, when incorporated into fruit-based (raspberry or pineapple) or dairy-based (Greek yogurt, cookies and cream, or chocolate with brownie) ice creams is capable of maintaining its viability above 10^7^ CFU/mL during 90 days of storage at −18 °C. This probiotic also maintained its anti-*H. pylori* activity in all ice cream bases; nevertheless, *H. pylori* inhibition was strong in dairy-based ice cream and a mild in fruit ice cream. Both fruit and dairy-based ice creams supplemented with *L. fermentum* UCO-979C were able to maintain the immunomodulatory activity of the probiotic on AGS cells, which produced the cytokine TGF-β_1_. The probiotic strain recovered from Greek yogurt ice cream caused a production of TGF-β1 close to that of the control, even at a concentration below 10^5^ CFU/mL after storage at −18 °C for 90 days. On the other hand, the addition of the *L. fermentum* UCO-979C strain in ice creams did not significantly modify the physicochemical properties of ice cream when compared with control ice creams, and it does not negatively affect the manufacture or nutritional composition of the ice creams.

These data show that the *L. fermentum* UCO-979C strain has a great potential in the production of dairy-based and fruit-based probiotic ice creams. Furthermore, considering that the functional properties of the probiotic strain are better in dairy-based ice creams, they can be considered more suitable candidates for incorporation in the probiotic biomass.

## Figures and Tables

**Table 1:** Viability of the probiotic strain *L. fermentum* UCO-979C in the different ice cream bases after 90 days of storage at −18 °C.

Time (Days)	15		30		60		90	
	Mean	SD	Mean	SD	Mean	SD	Mean	SD
Chocolate with brownie	8.917 ^a^	0.007	8.890 ^a^	0.009	8.812 ^b^	0.018	8.623 ^c^	0.031
Raspberry	7.915 ^a^	0.011	7.874 ^ab^	0.016	7.828 ^b^	0.024	7.855 ^b^	0.006
Greek yogurt	8.920 ^a^	0.024	8.909 ^a^	0.005	8.828 ^b^	0.009	8.840 ^b^	0.055
Cookies and cream	8.908 ^a^	0.005	8.904 ^a^	0.002	8.876 ^ab^	0.014	8.844 ^b^	0.010
Pineapple	8.021 ^a^	0.008	7.912 ^b^	0.013	7.890 ^b^	0.009	7.869 ^b^	0.020

Means with different superscript letters are significantly different (*p* < 0.05). SD means standard deviation.

**Table 2:** In vitro anti-*H. pylori* activity of the probiotic strain *L. fermentum* UCO-979C in the different ice cream bases at 90 days of storage at −18 °C.

Factor	MRS + Probiotic Bacteria (mm)	MRS without Probiotic Bacteria (mm)	Supernatant (mm)	Pellet (mm)	Saline Solution (mm)	Probiotic Inhibition Halo (mm) (a)	Supernatant Inhibition Halo (mm) (b)
Chocolate with brownie	4.5 ^1^	3	5.4	s/n	s/n	1.5 (+)	2.4 (++)
Greek yogurt	4.5	3	5.8	s/n	s/n	1.8 (+)	2.8 (++)
Cookies and cream	4.5	3	5.6	s/n	s/n	1.5 (+)	2.6 (++)
Raspberry	3.5	3	4.3	s/n	s/n	0.5 (+/−)	1.3 (+)
Pineapple	3.3	3	4	s/n	s/n	0.3 (+/−)	1 (+)
*L. fermentum*^2^ UCO-979C	5	3	5.1	s/n	s/n	2 (+)	2.1 (++)

Inhibition size: (>5 mm = +++) very strong inhibition; (5 mm and >2 mm = ++) strong inhibition; (2 mm and >1 mm = +) mild inhibition; and (≤1 mm = +/−) without inhibition (s/n) [21]. The halo of *H. pylori* J99 caused by the probiotic *L. fermentum* UCO-979C was calculated by subtracting the halo obtained with MRS medium without probiotic bacteria from the halo obtained with MRS medium with bacteria (a) or by subtracting the halo obtained with MRS medium without bacteria from the halo obtained with the supernatant (b). ^1^ Average of replicates. ^2^ Lyophilized.

**Table 3:** Chemical compositions in the control and probiotic *L. fermentum* UCO-979C fruit-based ice creams.

Fruit-Based Ice Creams						
	Raspberry			Pineapple		
Analysis	Probiotic	Control	*p* (Value)	Probiotic	Control	*p* (Value)
Ash %	0.165 ± 0.007	0.120 ± 0.014	0.0565	0.140 ± 0.014	0.095 ± 0.007	0.0565
Fat %	0.100 ± 0.000	0.100 ± 0.000	-	0.150 ± 0.071	0.065 ± 0.021	0.2450
Fiber %	0.980 ± 0.127	1.015 ± 0.007	0.7352	1.700 ± 0.141	1.475 ± 0.050	0.1677
Moisture %	76.820 ± 0.382	77.250 ± 0.396	0.3841	72.460 ± 0.580	74.335 ± 0.587	0.0847
Protein %	0.455 ± 0.021	0.305 ± 0.021	0.0194 *	0.270 ± 0.085	0.260 ± 0.071	0.9098
Sugar (g)	19.246 ± 0.024	19.238 ± 1.197	0.9946	23.920 ± 0.981	21.592 ± 0.233	0.0823
pH %	3.135 ± 0.007	3.420 ± 0.028	0.0052 *	3.670 ± 0.014	3.990 ± 0.014	0.0019 *
Lactic Acid %	0.868 ± 0.032	0.733 ± 0.015	0.0428 *	0.307 ± 0.004	0.259 ± 0.010	0.0253 *

Means with (*) are significantly different (*p* < 0.05).

**Table 4:** Chemical compositions in the control and probiotic ice cream *L. fermentum* UCO-979C, of dairy-based ice creams.

	Dairy-Based Ice Creams								
	Chocolate with Brownie			Cookies and Cream			Greek Yogurt		
Analysis	Probiotic	Control	*p* (Value)	Probiotic	Control	*p* (Value)	Probiotic	Control	*p* (Value)
Ash %	1.550 ± 0.028	1.490 ± 0.014	0.1153	1.100 ± 0.000	1.060 ± 0.014	-	0.960 ± 0.014	0.920 ± 0.014	0.1056
Fat %	6.100 ± 0.283	4.400 ± 0.283	0.0266 *	3.200 ± 0.283	2.550 ± 0.212	0.1215	1.100 ± 0.000	0.650 ± 0.071	0.0704
Fiber %	0.860 ± 0.000	0.850 ± 0.014	-	1.285 ± 0.035	1.355 ± 0.035	0.1863	0.550 ± 0.042	0.625 ± 0.007	0.1325
Moisture %	60.655 ± 3.331	57.250 ± 1.004	0.3005	59.315 ± 0.035	57.895 ± 2.044	0.5056	63.930 ± 0.127	65.265 ± 0.007	0.0045 *
Protein %	6.075 ± 0.050	6.445 ± 0.304	0.2315	4.855 ± 0.149	4.850 ± 0.057	0.9685	4.195 ± 0.219	4.105 ± 0.163	0.6868
Sugar (g)	19.159 ± 0.172	20.829 ± 0.284	0.0192 *	20.577 ± 0.399	19.971 ± 1.594	0.6537	19.180 ± 1.318	21.441 ± 0.024	0.2489
pH %	6.545 ± 0.007	6.605 ± 0.007	0.0136 *	6.780 ± 0.028	6.980 ± 0.014	0.0123 *	5.565 ± 0.021	5.655 ± 0.021	0.0513
Lactic Acid %	0.291 ± 0.032	0.244 ± 0.031	0.2724	0.175 ± 0.000	0.090 ± 0.000	-	0.551 ± 0.031	0.514 ± 0.032	0.3537

Means with (*) are significantly different (*p*< 0.05).

**Table 5:** Color parameters variables in the control and probiotic ice creams supplemented with *L. fermentum* UCO-979C.

		Chocolate with Brownie		Greek Yogurt		Cookies and Cream		Pineapple		Raspberry	
	Time (Days)	Ice Cream Control	Ice Cream Probiotic	Ice Cream Control	Ice Cream Probiotic	Ice Cream Control	Ice Cream Probiotic	Ice Cream Control	Ice Cream Probiotic	Ice Cream Control	Ice Cream Probiotic
L	15	29.29 b	33.36 a	68.64 a	70.47 a	73.52 a	73.51 ab	63.49 a	64.40 a	33.55 a	33.39 c
	30	29.44 b	33.36 a	66.51 b	70.53 a	73.45 a	73.53 a	62.40 b	63.45 b	33.57 a	34.56 b
	60	30.41 a	33.53 a	66.53 b	70.42 a	73.42 a	73.32 b	62.43 b	63.47 b	33.47 a	35.44 a
	90	30.48 a	33.42 a	66.59 b	69.52 b	73.41 a	73.47 ab	61.50 b	62.50 c	33.94 ab	35.51 a
C	15	10.67 b	17.69 b	5.08 b	4.31 b	10.77 b	11.90 b	21.09 c	18.37 b	37.64 b	36.20 b
	30	12.08 a	18.37 a	5.15 b	5.59 a	11.41 b	12.79 b	19.23 a	18.33 a	37.40 b	37.53 c
	60	11.95 a	17.65 b	5.14 b	5.56 a	12.22 a	13.19 a	19.19 b	17.45 b	38.39 a	38.40 b
	90	12.02 a	17.68 b	5.74 a	5.70 a	12.19 a	13.06 a	19.24 b	17.41 b	38.47 a	39.49 a
h	15	36.82 b	40.79 c	59.94 a	53.23 ab	127.67 a	118.18 b	105.17 b	107.56 c	77.05 b	77.83 a
	30	44.98 a	42.80 b	60.67 a	53.95 a	124.97 b	115.76 c	106.12 a	107.76 bc	76.74 b	76.99 c
	60	44.88 a	45.05 a	61.23 a	51.33 b	121.89 c	119.41 a	106.14 a	108.32 ab	76.99 b	77.32 bc
	90	45.00 a	45.05 a	52.33 b	51.39 b	121.92 c	119.10 a	106.61 a	^a^ 108.85 a	77.35 a	77.60 ab
ΔE	15	-	-	-	-	-	-	-	-	-	-
	30	2.150	0.934	2.133	1.287	0.833	1.041	2.182	0.958	0.314	1.848
	60	2.326	1.330	2.120	1.272	1.856	1.331	2.198	1.337	0.762	3.025
	90	2.422	1.320	2.273	1.694	1.830	1.182	2.762	2.174	1.329	3.926

Lower case letters next to the means in the same column indicate significant differences (*p* < 0.05). L*C*h* system: (L) brightness; (C) chroma; (h) hue.

## Supplementary Materials

The following supporting information can be downloaded at: https://www.mdpi.com/article/10.3390/foods11030333/s1, Figure S1: Fall in the first drop of different matrices of ice creams; Figure S2: Melting speed of different matrices of ice creams.
