# Supplementary material for: Incorporation of Limosilactobacillus fermentum UCO-979C with Anti-Helicobacter pylori and Immunomodulatory Activities in Various Ice Cream Bases
Source: Foods. 2022 Jan 25;11(3):333. doi: 10.3390/foods11030333 (PMC8834266; doi:10.3390/foods11030333)
Supplement: Supplementary file 1 [file foods-11-00333-s001.zip › foods-1469883-supplementary.pdf]

Supplementary Materials

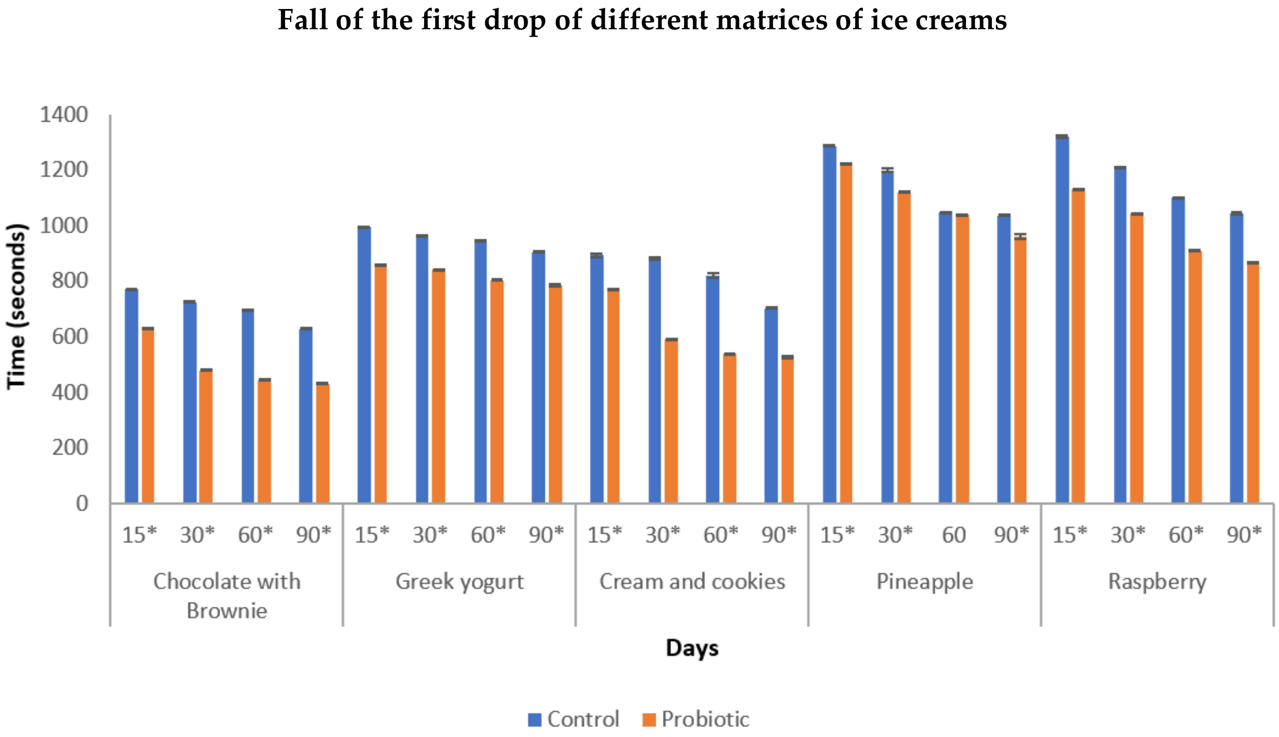

Figure S1. Fall of the first drop of ice cream in the different matrices of control ice creams and probiotic *L. fermentum* UCO-979C supplemented ice cream matrices. Means with (*) indicates significant differences ($p < 0.05$).

Melting speed of different matrices of ice creams

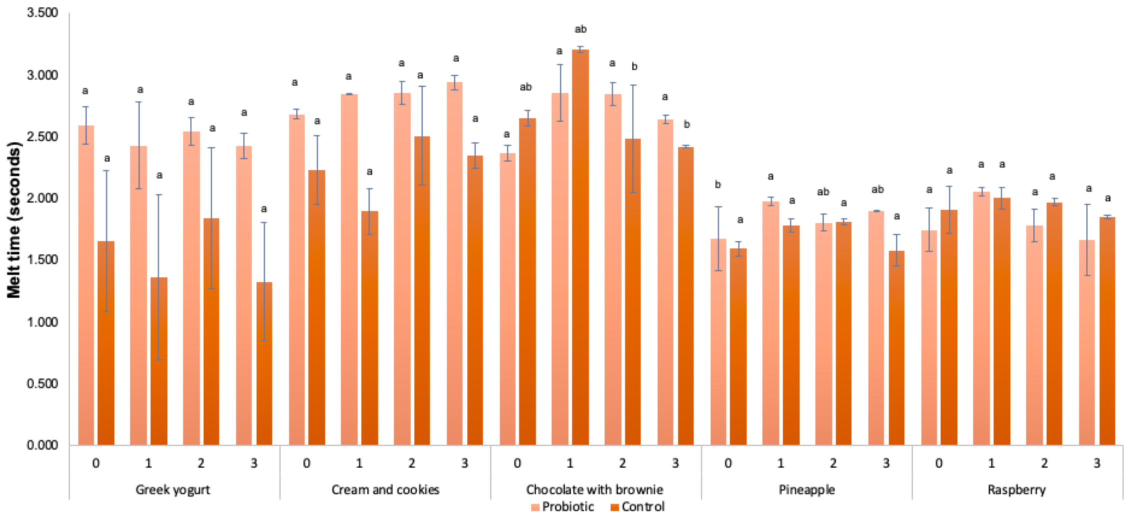

Figure S2. Melting speed of control ice cream (without probiotic) and probiotic ice creams supplemented with probiotic *L. fermentum* UCO-979C. Means with different letters are significantly different ($p < 0.05$).
